# Endovascular treatment of a craniocervical junction dural arteriovenous fistula associated with lateral medullary syndrome: A case report

**DOI:** 10.1016/j.radcr.2025.05.021

**Published:** 2025-05-30

**Authors:** Masaaki Kubota, Yosuke Tajima, Yoshinori Higuchi

**Affiliations:** a Department of Neurological Surgery, Chiba University Graduate School of Medicine, Chiba, Chiba Prefecture, Japan; b Comprehensive Stroke Center, Chiba University Hospital, Chiba, Chiba Prefecture, Japan

**Keywords:** Craniocervical junction, Dural arteriovenous fistula, Lateral medullary syndrome, Endovascular treatment, Brainstem infarction

## Abstract

Intracranial dural arteriovenous fistulas (DAVFs) with drainage into the perimedullary veins have been reported to cause brainstem and spinal hemorrhages, subarachnoid hemorrhages, and progressive myelopathy. However, there have been no reports of craniocervical junction arteriovenous fistulas (CCJ-AVFs) complicated by lateral medullary syndrome (LMS) and subsequently treated. We present a case successfully treated with transvenous and transarterial embolization. A 67-year-old man presented with headache and dizziness was diagnosed with left LMS based on diffusion-weighted MRI. MRA ruled out vertebral artery dissection and posterior inferior cerebellar artery occlusion but suggested an arteriovenous shunt at the CCJ, which digital subtraction angiography confirmed as a DAVF fed by the radiculomeningeal artery with drainage into the anterior lateral spinal vein and deep brainstem veins. To minimize embolic complications, transvenous embolization with coils was performed first, followed by transarterial embolization with N-butyl cyanoacrylate. Postoperative MRI showed resolution of venous engorgement, and the patient was discharged without additional neurological deficits. This case highlights the potential role of AVF-induced venous engorgement in brainstem infarction and underscores the importance of early diagnosis and individualized treatment. A combined transvenous and transarterial approach can effectively control ascending venous outflow while minimizing procedural risks.

## Introduction

Dural arteriovenous fistulas (DAVFs) at the craniocervical junction (CCJ) account for 1%-2% of intracranial and spinal AVFs, with 37% classified as DAVFs [1]. CCJ-AVFs may present acutely with subarachnoid or intramedullary hemorrhage or more insidiously with venous congestion-related progressive myelopathy or brainstem dysfunction [2,3]. Treatment options include surgical disconnection or endovascular embolization of AVF [4]. While surgical approaches provide direct visualization, they are invasive and associated with risks such as infection and cerebrospinal fluid leakage [5]. Endovascular therapy offers a less invasive alternative but carries risks of embolic migration and catheter-related complications [6]. We report a case of CCJ-AVF associated with lateral medullary syndrome (LMS), successfully treated with transvenous and transarterial embolization.

## Case report

**Fig. 1:**
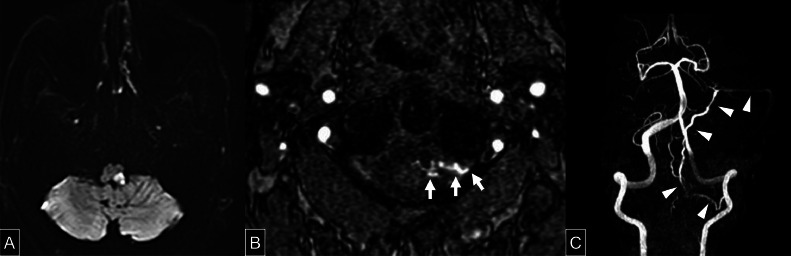
(A) Axial diffusion-weighted MRI showing a lesion with restricted diffusion in the left lateral medulla. (B) Axial MR angiographic image at the level of the second cervical vertebra. A white arrow indicates abnormal vessels extending from the epidural space toward the spinal cord. (C) MR angiographic image with the bilateral internal and external carotid arteries digitally subtracted. A white arrowheads indicates a vessel arising from the left vertebral artery, coursing medially and then ascending to the superior petrosal sinus.

A 67-year-old man presented with headache and dizziness and was diagnosed with left LMS. MRI revealed high signal intensity on diffusion-weighted imaging (DWI) in the left lateral medulla. (Fig. 1A) LMS, also known as Wallenberg syndrome, is caused by ischemia in the dorsolateral medulla and typically presents with ipsilateral facial and contralateral body pain and temperature sensory loss, dysphagia, vertigo, ataxia, and Horner’s syndrome. MRA showed no vertebral artery dissection or posterior inferior cerebellar artery occlusion but raised suspicion of a shunting lesion at the CCJ. (Fig. 1Fig. 1, Fig. 1) Following acute-phase stroke management and rehabilitation, the patient was referred to our institution. At the time of presentation to our institution, neurological examination revealed dysphagia and sensory disturbances in the right upper and lower limbs as sequelae of LMS. These sequelae, including bulbar dysfunction and sensory deficits, are common and can persist chronically, affecting quality of life.

**Fig. 2:**
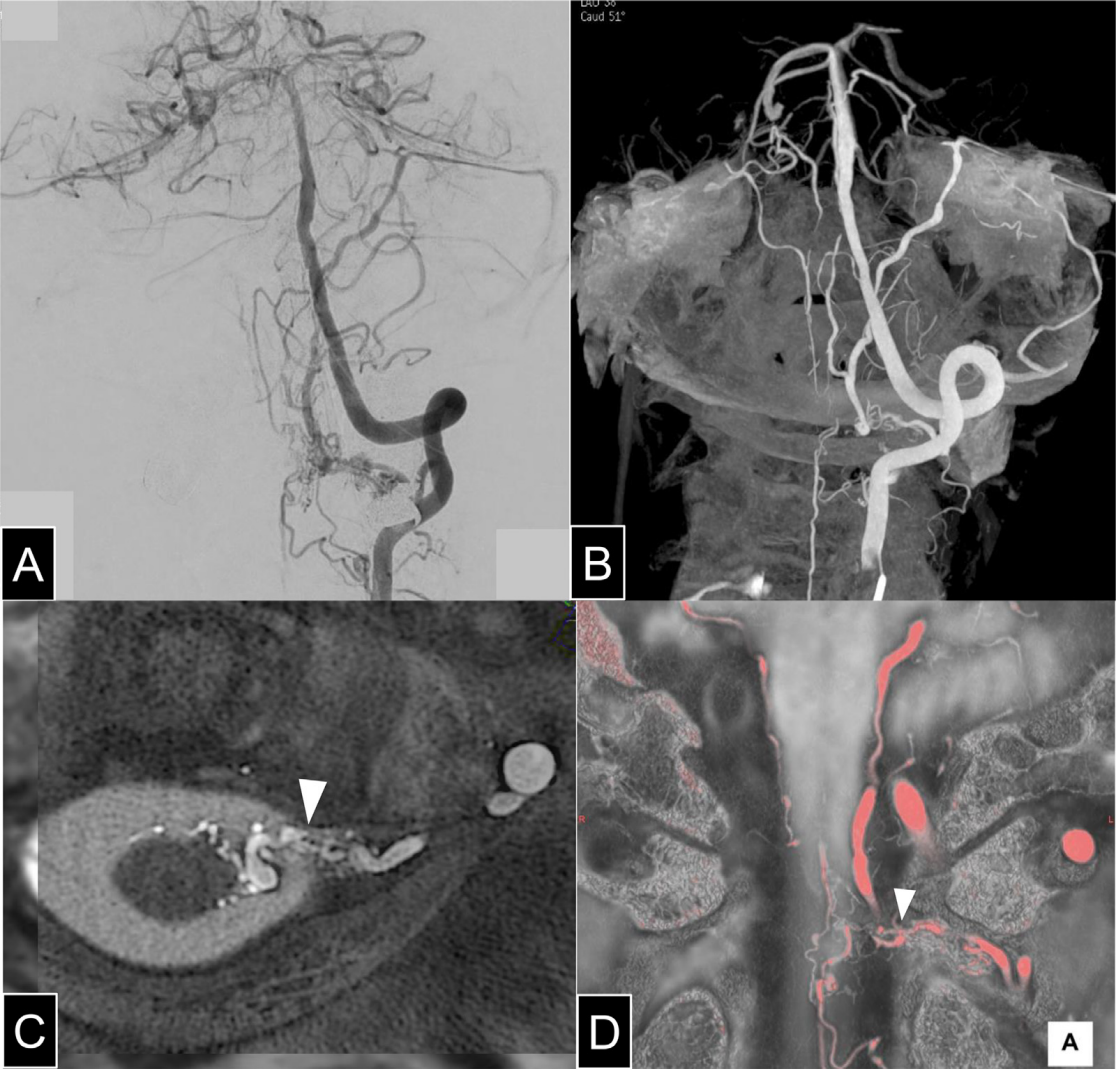
(A) An anterior-posterior view of left vertebral angiography demonstrates a radiculomeningeal artery arising from the V3 segment and extending medially, forming an arteriovenous fistula with venous drainage observed both superiorly and inferiorly. Image quality in the surrounding area was partially degraded due to digital subtraction artifacts caused by a dental metal crown. (B) 3D rotational angiography of the left vertebral artery demonstrates a radiculomeningeal artery (white arrow) forming a fistulous connection with the anterior lateral spinal vein, with clear visualization of the venous drainage routes. Bilateral lateral medullary veins and lateral pontine veins are visualized, with marked dilation observed on the left side. The venous drainage proceeds via the superior petrosal sinus and petrosal vein into the basal vein of Rosenthal. (C) Axial fused image of heavily T2-weighted MRI and 3D rotational angiography of the left vertebral artery. The white arrowhead indicates the shunt point. Based on the vessel caliber change observed on the dura mater, the lesion was diagnosed as a dural arteriovenous fistula (DAVF). (D) Coronal fused image of heavily T2-weighted MRI and 3D rotational angiography of the left vertebral artery, with vascular structures rendered in red. The white arrowhead indicates the shunt point located on the dura mater.

DSA identified a CCJ-AVF with feeders from the left C2 radiculomeningeal artery (RMenA) draining into the anterior lateral spinal vein (ALSV), lateral medullary vein (LMV), lateral pontine vein, basal vein of Rosenthal, superior petrosal sinus (SPS), and the anterior spinal vein (ASV), suggesting perimedullary venous drainage. (Fig. 2A and B) Fusion imaging with heavily T2-weighted MRI and 3D rotational angiography localized the fistula to the dura, confirming the DAVF diagnosis.(Fig. 2Fig. 2, Fig. 2) AVF-induced dilation of brainstem veins likely caused compression of arteries supplying the brainstem, leading to infarction. In addition to mechanical compression of the arterial supply by AVF-induced venous dilation, venous congestion may have contributed to impaired outflow and secondary venous infarction. Given the potential risk of hemorrhage and venous congestion-related neurological symptoms, and considering the patient's preference, endovascular treatment was selected.

Because direct embolization via C2 RMenA posed a risk of N-butyl cyanoacrylate (NBCA) (Histoacryl, B.Braun, Melsungen, Germany) migration into deep brainstem veins, we opted for a staged approach. Initially, a transvenous coil embolization was performed to occlude ALSV via SPS and LMV. Then, transarterial embolization was performed via C2 RMenA using NBCA diluted with ethyl ester of iodinated poppy-seed oil fatty acid (Lipiodol, Guerbet Japan, Tokyo, Japan).

**Fig. 3:**
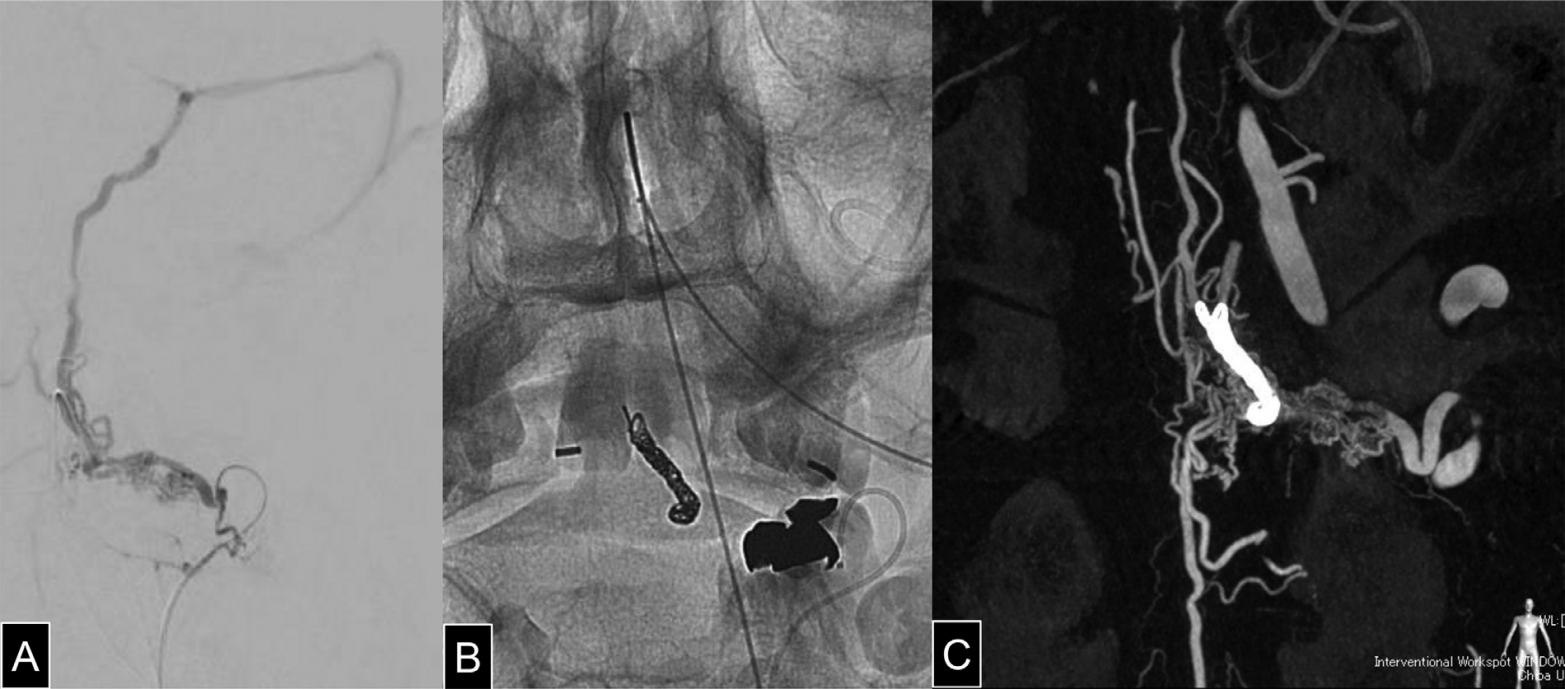
(A) Selective angiography of the radiculomeningeal artery arising from the left vertebral artery demonstrates venous drainage reaching the anterior lateral spinal vein via the internal jugular vein and superior petrosal sinus. (B) The left anterior lateral spinal vein was embolized with 7 coils via a transvenous approach. (C) Cone-beam CT performed via the left vertebral artery after coil embolization reveals persistent perimedullary venous enhancement in the arterial phase, indicating incomplete occlusion of the shunt.

**Fig. 4:**
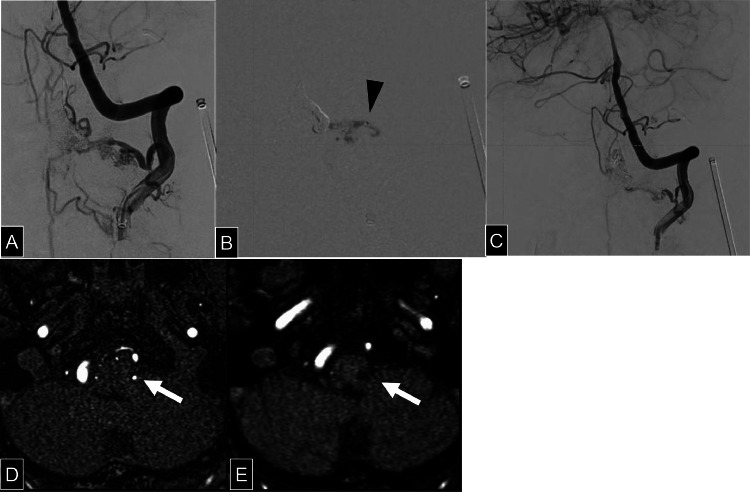
(A and B) Left vertebral angiography following coil embolization of the anterior lateral spinal vein is shown (A). A microcatheter was advanced into the C2 radiculomeningeal artery, through which 50% diluted N-butyl cyanoacrylate was administered (B). The black arrowhead indicates the tip of the microcatheter. (C) Following arterial embolization angiography of the left vertebral artery demonstrates disappearance of the ascending draining vein previously observed preoperatively. Minimal residual flow into the anterior spinal vein was noted during the arterial phase. (D and E) An infarct in the left lateral medulla is observed on diffusion-weighted imaging (A), and a corresponding lateral medullary vein (LMV) is visualized at the same location on axial MR angiography (MRA) (white arrow, D). On postoperative MRA, the signal from the LMV observed preoperatively is no longer visualized (white arrow). The hypointensity lesion in the left lateral medulla represents changes consistent with chronic infarction.

Surgical Procedure: Under general anesthesia, a 4Fr guiding sheath (Fubuki dilator kit, Asahi Intecc Co Ltd, Aichi, Japan) and a 3.2/3.4Fr intermediate catheter (Guidepost, Tokai Medical Products, Aichi, Japan) were introduced via the right femoral artery. A 1.7Fr microcatheter (Breakthrough, Boston Scientific, Marlborough, MA) was advanced into the left C2 RMenA. (Fig. 3A) Venous access was obtained via the left femoral vein, where a 4Fr guiding sheath was placed in the left internal jugular vein. A 3.2/3.4Fr intermediate catheter and a 1.6Fr microcatheter (Headway Duo, MicroVention-Terumo, Aliso Viejo, CA) were navigated through the SPS and LMV into the ALSV. Transvenous coil embolization of the ALSV was performed using 7 coils. (Fig. 3Fig. 3, Fig. 3) Subsequently, a 2.2/1.3Fr flow-directed catheter (DeFrictor Nano, Medico's Hirata, Osaka, Japan) was advanced as close to the fistulous connection as possible, and 0.25 mL of 50% diluted NBCA was injected. Postembolization DSA confirmed occlusion of the ascending venous drainage route, while minimal residual flow into the ASV persisted (Fig. 4A-C).

Post operative MRI showed no new infarcts, and venous engorgement in the brainstem resolved.(Fig. 4D) The patient was discharged without new neurological deficits. Given the persistence of ASV drainage, there remains a risk of developing myelopathy in the future; therefore, regular outpatient follow-up is ongoing. Follow-up DSA performed at 6 months and MRI at 1 year postoperatively revealed no progression of the AVF, and no new abnormalities. Although 2 years have passed since surgery, the patient remains asymptomatic to date.

## Discussion

CCJ-AVF is a rare condition that requires early diagnosis and treatment to prevent permanent neurological deficits. Surgical approaches allow for direct occlusion of the shunt, and the use of intraoperative DSA and neurophysiological monitoring can enhance procedural safety. Additionally, some reports suggest that surgical intervention provides higher curative rates than endovascular treatment [7]. However, surgical approaches involve procedural invasiveness, with complex access to the ventral aspect of the spinal cord, and are associated with risks of postoperative infection and cerebrospinal fluid leakage. In contrast, endovascular treatment is a minimally invasive procedure that can be performed under local anesthesia. Nevertheless, it carries risks such as migration of embolic materials leading to cerebral or spinal infarction, secondary thrombosis associated with the use of liquid embolic agents, and difficulties in catheter removal. However, recent advancements in endovascular devices and angiographic imaging technology have contributed to improved treatment outcomes, with promising results being reported in the literature [8].

In endovascular treatment, the primary strategy generally involves embolization of the shunt point via either a transarterial or transvenous approach. In transarterial embolization, particularly in cases with a high-flow feeder, there is a risk of embolic material migrating beyond the shunt point to the distal venous side, potentially leading to delayed venous occlusion and subsequent venous infarction, which may result in neurological deficits [9]. In the present case, to prevent embolic material from migrating into the venous system, we first performed transvenous coil occlusion of the draining vein, followed by transarterial embolization, achieving a favorable outcome. However, it is important to note that prior transvenous coil embolization may lead to partial occlusion of the draining vein, which can result in early retrograde reflux of the embolic material during subsequent transarterial embolization, thereby increasing the risk of unintended occlusion of the parent artery. The integration of angiographic imaging with other modalities such as MRI facilitated a detailed evaluation of vascular anatomy and precise identification of the shunt point, contributing to the optimization of our treatment strategy.

This case represents a rare instance of CCJ-AVF complicated by LMS. To the best of our knowledge, no similar cases have been previously reported in the literature. According to prior studies, LMS is most commonly caused by ipsilateral vertebral artery (VA) stenosis or occlusion in approximately 67% of cases, posterior inferior cerebellar artery (PICA) lesions in about 10%, and VA or PICA dissections in 14%-33% of cases [10]. In the present case, no apparent stenosis, occlusion, or dissection of the VA or PICA was identified. In previously reported cases of intracranial DAVFs associated with brainstem infarction, the proposed mechanisms have included venous hypertension, arterial steal phenomenon, and direct compression by dilated venous structures [11]. In our case, preoperative MRA revealed a dilated vein at the lateral medulla corresponding to the infarcted region. The lateral medulla is typically perfused by perforating branches of the lateral arteries originating from either the VA or PICA, which directly penetrate and supply this region [12]. The observed venous dilation, associated with the AVF, was presumed to have caused mechanical compression of these perforating arteries, resulting in focal ischemia. Following treatment, the dilated vein was no longer visualized. These findings suggest that direct venous compression by the AVF-associated dilated vein was the most likely mechanism of infarction. However, venous engorgement itself may have also contributed to impaired venous outflow and venous hypertension, which could have played a role in the pathogenesis of the infarct. Thus, both mechanical arterial compression and venous congestion should be considered as potential mechanisms.

This case highlights the importance of multimodal imaging, particularly MRI fusion imaging, which enables precise anatomical assessment of vascular structures. By integrating MRI and DSA, we were able to more accurately localize the shunt point, optimize catheter positioning, and minimize the risk of embolic migration—ultimately improving the safety and efficacy of the procedure.

## Conclusion

AVF-induced venous engorgement likely caused direct compression, contributing to the infarction. This case underscores the importance of detailed vascular imaging for diagnosis and individualized treatment strategies to optimize patient outcomes. This report presents a rare case of CCJ-AVF associated with LMS, successfully treated with a staged endovascular approach. Transvenous coil embolization followed by transarterial NBCA embolization prevented embolic complications and led to favorable clinical outcomes. Given the potential for progressive neurological deterioration, early recognition and individualized treatment strategies are essential for optimal management of CCJ-AVFs.

## Patient consent

Written informed consent was obtained from the patient for the publication of this case report, including all associated images and information. The authors confirm that the consent documentation is retained in accordance with institutional and journal requirements.

## References

[1] Hiramatsu M., Sugiu K., Ishiguro T., Kiyosue H., Sato K., Takai K. (2018). Angioarchitecture of arteriovenous fistulas at the craniocervical junction: a multicenter cohort study of 54 patients. J Neurosurg.

[2] Hiramatsu M., Sugiu K., Hishikawa T., Haruma J., Tokunaga K., Date I. (2014). Epidemiology of dural arteriovenous fistula in Japan: analysis of Japanese registry of Neuroendovascular Therapy (JR-NET2). Neurol Med Chir (Tokyo).

[3] Endo T., Shimizu H., Sato K., Niizuma K., Kondo R., Matsumoto Y. (2014). Cervical perimedullary arteriovenous shunts: a study of 22 consecutive cases with a focus on angioarchitecture and surgical approaches. Neurosurgery.

[4] Willinsky R., Lasjaunias P., Terbrugge K., Hurth M. (1990). Angiography in the investigation of spinal dural arteriovenous fistula. A protocol with application of the venous phase. Neuroradiology.

[5] Takai K. (2019). Update on the diagnosis and treatment of arteriovenous fistulas at the Craniocervical junction: a systematic review of 92 cases. J Neuroendovasc Ther.

[6] Zhao J., Xu F., Ren J., Manjila S., Bambakidis NC. (2016). Dural arteriovenous fistulas at the craniocervical junction: a systematic review. J Neurointerv Surg.

[7] Wang J.Y., Molenda J., Bydon A., Colby G.P., Coon A.L., Tamargo R.J. (2015). Natural history and treatment of craniocervical junction dural arteriovenous fistulas. J Clin Neurosci.

[8] Lee S., Kubota M., Tajima Y., Kojima I., Higuchi Y. (2024). Transarterial embolization of radicular arteriovenous fistula at the craniocervical junction. Radiol Case Rep.

[9] Qi X., Lv L., Han K., Xu Z., Mei Q., Chen H. (2014). Analysis of the embolization spinal dural arteriovenous fistula and surgical treatments on 52 cases of the patients. Int J Clin Exp Med.

[10] Kim J.S., Caplan LR. (2016). Clinical stroke syndromes. Front Neurol Neurosci.

[11] Satoh M., Kuriyama M., Fujiwara T., Tokunaga K., Sugiu K. (2005). Brain stem ischemia from intracranial dural arteriovenous fistula: case report. Surg Neurol.

[12] Vlašković T., Brkić B.G., Stević Z., Kostić D., Stanisavljević N., Marinković I. (2022). Anatomic and MRI bases for medullary infarctions with patients' presentation. J Stroke Cerebrovasc Dis.

